# Krüppel-Like Factor 2 Is a Gastric Cancer Suppressor and Prognostic Biomarker

**DOI:** 10.1155/2023/2360149

**Published:** 2023-02-23

**Authors:** Xi-mei Li, Sheng-juan Hu, Jian-fang Liu, Mei-juan Ma, Li-meng Du, Fei-hu Bai

**Affiliations:** ^1^School of Clinical Medicine, Ningxia Medical University, Yinchuan, Ningxia 750004, China; ^2^Department of Gastroenterology, People's Hospital of Ningxia Hui Autonomous Region, Yinchuan, Ningxia 750002, China; ^3^Department of Gastroenterology, The Second Affiliated Hospital of Hainan Medical University, The Gastroenterology Clinical Medical Center of Hainan Province, Haikou 570216, China

## Abstract

Gastric cancer (GC) is a common digestive tract tumor. Due to its complex pathogenesis, current diagnostic and therapeutic effects remain unsatisfactory. Studies have shown that KLF2, as a tumor suppressor, is downregulated in many human cancers, but its relationship and role with GC remain unclear. In the present study, KLF2 mRNA levels were significantly lower in GC compared to adjacent normal tissues, as analyzed by bioinformatics and RT-qPCR, and correlated with gene mutations. Tissue microarrays combined with immunohistochemical techniques showed downregulation of KLF2 protein expression in GC tissue, which was negatively correlated with patient age, T stage, and overall survival. Further functional experiments showed that knockdown of KLF2 significantly promoted the growth, proliferation, migration, and invasion of HGC-27 and AGS GC cells. In conclusion, low KLF2 expression in GC is associated with poor patient prognosis and contributes to the malignant biological behavior of GC cells. Therefore, KLF2 may serve as a prognostic biomarker and therapeutic target in GC.

## 1. Introduction

Gastric cancer (GC) ranks fifth among common cancers and third among cancer-related causes of death [1]. China is a high-incidence area for GC [2]. Despite great improvements in medical equipment and treatment, overall survival remains low in GC patients, and many patients are in advanced stages once diagnosed [3, 4]. Increasing evidence suggests that *H. pylori* infection is a major risk factor for the development of GC, but the incidence of *H. pylori*-related GC is significantly reduced due to vaccination of patients or taking drugs to treat *H. pylori* infection, while the number of GC patients due to genetic variants is increasing [5]. Pereira et al. showed that transcriptional alterations and copy number variations (CNV) of GSDMC in GC are associated with stronger tumor aggressiveness [6]. In addition, an increasing number of studies have found that abnormal genomic expression in GC patients is associated with poor patient prognosis [7–9]. However, due to genetic heterogeneity and complex patterns of genetic span across different tumor stages, so far, no specific biomarker has been able to accurately diagnose or predict the prognosis of GC [10]. Many patients lack specific diagnostic biomarkers and targeted therapies, resulting in significantly reduced survival times. Therefore, it is important to explore new diagnostic and prognostic biomarkers as well as to develop targeted therapeutics for the diagnosis and treatment of GC.

Krüppel-like factor2 (KLF2) is an important member of the Krüppel-like factor (KLF) family, which plays an antitumor role in many cancers [11]. KLF family members are involved in cell differentiation, proliferation, migration, and pluripotency as transcriptional regulators [12–14]. KLF has been reported to be prone to genomic changes. Yin et al. showed that the downregulation of KLF2 expression in nonsmall cell lung cancer is associated with a poor prognosis for patients [15]. In GC, KLF2 is inhibited by the regulation and expression of long chain noncoding RNA (lncRNA) and microRNA (miRNA), thus promoting the progress of GC [16, 17]. A recent study showed that downregulation of KLF2 expression in human cancers is caused by epigenetic silencing of histone methyltransferase EZH2 [11]. These studies suggest that KLF2 as a tumor suppressor gene is closely related to tumor development. However, the relationship between KLF2 and GC and its effect on GC has not been fully elucidated. Therefore, it is worth further revealing the relationship between KLF2 and GC, its clinical characteristics, and its role.

In recent years, bioinformatics analysis of large sample data combined with clinical studies and molecular experiments to identify reliable genetic biomarkers is another important means to study tumor pathogenesis and discover new therapeutic targets. Wei et al. demonstrated that miR-486-5p is a tumor suppressor of GC and inhibits gastric cancer cell growth and migration by downregulating fibroblast growth factor 9, through the TCGA database and cell experiments [18]. In addition, Chu et al. used bioinformatics techniques combined with clinical data to analyze the expression of thrombospondin-2 (TSP2) in GC tissues and its correlation with clinicopathological features and clinical prognosis of GC patients, indicating that TSP2 is a potential marker and therapeutic target for the prognosis of GC patients. In vitro experiments further demonstrated that TSP2 promoted the proliferation and migration of HGC-27 and AGS GC cell lines by inhibiting the VEGF/PI3K/AKT signaling pathway [19]. Therefore, the aim of this study was to investigate the mechanism of KLF2 expression in GC, the correlation of clinical features, and the effect on the biological function of GC cells by using bioinformatics techniques and molecular biology experiments.

## 2. Materials and Methods

### 2.1. Data Collection and Different Expression Analysis

Tumor Immune Estimation Resource 2.0 (TIMER2.0) (https://timer.cistrome.org/) database was used to analyze differential expression of KLF2 mRNA levels in different human tumors [20] and focused on KLF2 expression in GC. Next, The Cancer Genome Atlas (TCGA) program (https://www.cancer.gov/tcga) [21] and The Gene Expression Profiling Interactive Analysis 2 (GEPIA2, https://gepia2.cancer-pku.cn/) [22] were used to compare KLF2 expression levels in gastric cancer and adjacent normal tissues. Then, the Cancer Cell Line Encyclopedia (CCLE) database (https://portals.broadinstitute.org/ccle) was used to assess KLF2 expression levels in different cancer cell lines [23].

### 2.2. Genetic Changes and Prognostic Value Analysis

Using the cBio Cancer Genomics Portal (cBioPortal) online software (https://cbioportal.org), genomic alterations of KLF2 in GC were analyzed, including mutations in KLF2 and the proportion of data distribution and the frequency of changes (mutations and amplifications) of KLF1 gene in gastric cancer subtypes [24]. Also, the association between KLF2 gene alterations and overall survival, disease-free survival, and progression-free survival in GC patients was investigated to assess the prognostic value. Subsequently, we also analyzed copy number variation (CNV) of KLF2. In addition, the CNV module of Gene Set Cancer Analysis (GSCA; https://bioinfo.life.hust.edu.cn/GSCA/#/) was used to analyze the proportion of KLF2 heterozygous/homozygous and amplified/deleted in GC, the Spearman correlation between KLF2 mRNA expression and CNV, and survival differences between KLF2 CNV and wild type [25].

### 2.3. Collecting Tumor Tissue

Thirty GC patients (median age: 61 years; age: 27–75 years) who underwent surgical resection at People's Hospital of Ningxia Hui Autonomous Region from 2019 to 2021 and their adjacent normal gastric tissues were collected and immediately stored in liquid nitrogen. No patients received chemotherapy or radiotherapy prior to sample collection. Each participant or his/her authorized representative signed an informed consent form. This study was approved by the Ethics Committee of Ningxia Hui Autonomous Hospital (Approval No.: 2017–034) and conducted according to the World Medical Association Declaration of Helsinki.

### 2.4. Tissue Microarray and Immunohistochemistry

A chip (No.: HStmA180Su19) containing 86 adjacent noncancerous specimens and 88 gastric cancer tissues was purchased from Shanghai Biochip Limited company. The specimens were subjected to TNM pathological staging according to the 7th edition of the AJCC/UICC tumor lymph node metastasis (TNM) staging classification. The expression of KLF2 protein in the samples was detected by the immunohistochemical EnVision method. Following deparaffinization, hydration, antigen retrieval, and blocking endogenous peroxidase activity of the tissue microarray according to the kit instructions, the chip was incubated with KLF2 polyclonal antibody (PA5-40591, Invitrogen, USA) for 1 h. Afterwards, the chip was processed using the EnVision Rb Gt anti-Rb-HRP kit (K4003, Dako, Denmark) according to the manufacturer's instructions and visualized by DAB staining solution (Dako, Denmark) in the dark. Following rinsing in tap water, the chips were counterstained for chips using hematoxylin, dehydrated in a graded water-ethanol series, soaked in xylene, and finally sealed with neutral gum. Staining signals were visualized using a Nikon microscope (Tokyo, Japan) and statistically analyzed using ImageJ Pro (Media Cybernetics, Rockville, Maryland, United States). Quantitative analysis was performed independently by two pathologists who were blinded to the experimental groupings. The final score is obtained by multiplying the staining intensity and the percentage of positive tumor cells and ranges from 0 to 300. Staining intensity scores were 0 (negative), 1+ (weak), 2+ (moderate), and 3+ (strong). Percentage of positive tumor cells is 0–100%. A final score of <100% is considered low and >100% is considered high [26].

### 2.5. Cell Culture and Transfection

GC cell lines (HGC-27 and AGS) were purchased from American Type Culture Collection and cultured in RPMI-1640 (Gibco, USA) supplemented with 10% fetal bovine serum (FBS; Gibco) and 1% penicillin-streptomycin (Gibco) and grown at 37°C and 5% CO_2_. Digestion and passage were performed using 0.25% trypsin (Solarbio, Beijing, China) after cells reached 80–90% confluence.

KLF2 siRNA (si-KLF2) and its control (si-control) were designed and synthesized by Invitrogen (USA). When AGS and HGC-27 cells reached 80% confluency, the above siRNAs were transfected into GC cells using Lipofectamine® 2000 (Thermo Fisher Scientific, Inc., USA) according to the manufacturer's instructions. Six hours after transfection, the fresh serum-containing medium was replaced. The culture was continued for 48 h and cells were collected for CCK-8, colony formation, wound healing, transwell, RT-qPCR, and immunoblot analysis. The si-KLF2 sequence is as follows: si-KLF2: sense: UCAACAGCGGCUGGACUUTT, antisense: UAAGCCAGCACGCUGUGGAUTT.

### 2.6. Cell Counting Kit-8 (CCK-8) Assay

Transfected HGC-27 and AGS cells (1 × 10^4^ cells/well) were seeded into 96-well plates. Following incubation at 37°C and 5% CO_2_ for 24, 48, and 72 h, 10 *μ*l CCK-8 solution was added to each well and incubated for 2 h. Absorbance at 450 nm was measured using a microplate reader (BioTek Instruments, USA) [27].

### 2.7. Colony Formation Assay

Transfected AGS and HGC-27 cells (5 × 10^2^ cells/well) were seeded into 12-well plates and cultured for 14 days at 37°C in a 5% CO_2_ incubator to form macroscopic colonies, the medium was removed, and the cells were washed three times with PBS; then, the colonies were fixed with 4% paraformaldehyde for 20 minutes at room temperature and the colonies were stained with 0.1% crystal violet for 10 minutes. Images were taken under a light microscope, and the number of cell colonies >50 cells was calculated [27].

### 2.8. Wound Healing Assay

Transfected GC cells (3 × 10^5^ cells/well) were seeded into 24-well plates and cultured at 37°C and 5% CO_2_ for 24 h. When more than 90% confluent monolayers were formed, cell monolayers were scratched with a 100 *μ*l pipette tip and washed with PBS to remove floating cells. Subsequently, fresh serum-free medium was added to each well, and after 24 h of culture, images at 0 and 24 h after scratching were collected with a light microscope (Nikon, Japan), and then, wound healing rates were measured and calculated using ImageJ software. The formula for wound healing rate is as follows: [wound width (0 h) wound width (48 h)]/wound width (0 h)×100% [27].

### 2.9. Transwell Assay

Transfected AGS and HGC-27 cells (1 × 10^5^ cells/ml) were suspended in serum-free RPMI-1640 medium and seeded into the upper chamber precoated with Matrigel (BD Biosciences). Then, RPMI-1640 medium containing 10% FBS was added to the lower chamber of a 24-well plate and cultured for 24 h at 37°C in an incubator with 5% CO_2_. Remove the culture medium. Cells remaining in the upper chamber were gently removed with a cotton swab. Cells were rinsed three times with PBS, and then, cells were fixed with 4% paraformaldehyde for 20 min at room temperature and stained with 0.1% crystal violet for 10 min at room temperature. Under a light microscope, five fields were randomly selected to observe cells, acquire images, and calculate the number of migrating or invading cells [28].

### 2.10. Quantitative Real-Time Polymerase Chain Reaction (RT-qPCR)

Total RNA from tissues and cells was extracted using TRIzol reagent (Invitrogen, Carlsbad, CA, USA) and tested for concentration and purity of RNA. RNA was subsequently reverse transcribed to synthesize cDNA using the cDNA Reverse Transcription Kit (Thermo Fisher, Hudson, NH, USA). cDNA was amplified using the SYBR Green RT-qPCR kit (Thermo Fisher, Hudson, NH, USA) according to the supplier's guidelines to detect mRNA expression levels of KLF2. *β*-Actin was used as an internal normalization control, and relative expression levels of genes were calculated by the 2^−ΔΔCT^ method [29]. The primer sequences were as follows: KLF2-F: 5′-CTACACCAAGAGTTCGCATCTG-3′; KLF2-R: 5′-CCGTGGCTTTCGGTAGTG-3′; human *β*-actin-F1: 5′-GAGAGCTACGAGCTGCCTGA-3′; human *β*-actin-R1: 5′-CAGACAGCACTGTTGGCG-3′.

### 2.11. Western Blotting Analysis

Total protein was extracted by lysing cells with RIPA lysis buffer containing PMSF (Beyotime Biotechnology, Shanghai, China). Then, protein concentration was detected using a BCA protein assay kit (Beyotime Biotechnology, Shanghai, China). Protein samples (30 *μ*g) were separated with 10% SDS-PAGE. Subsequently, proteins were transferred to polyvinylidene fluoride (PVDF) membranes (Merck Millipore, Billerica, MA, USA). PVDF membranes were blocked with 5% skim milk for 1 h at room temperature. Afterwards, PVDF membranes were incubated overnight at 4°C with primary antibodies KLF2 (1 : 1000; Beyotime, China), E-cadherin (1 : 1000; Beyotime, China), N-cadherin (1 : 1000; Beyotime, China), Snail (1 : 1000; Beyotime, China), vimentin (1 : 1000; Beyotime, China), Twist (1 : 1000; Beyotime, China), and *β*-actin (Beyotime, China). On the following day, PVDF membranes were incubated with horseradish peroxidase-conjugatedanti-rabbit IgG secondary antibody (1 : 5000; Sigma-Aldrich, USA) and anti-mouse IgG secondary antibody (1 : 5000; Cell Signaling Technology, Inc., USA) for 1 h at room temperature. Finally, protein bands were visualized using the BeyoECL Plus kit (Beyotime Biotechnology, China) and Image Pro Plus 6.0 software (Media Control, Inc., USA) for analysis.

### 2.12. Statistical Analysis

All gene expression data were normalized by log2 transformation. Kaplan–Meier analysis and log-rank test were used for survival analysis. Cox regression models were used to analyze the relationship between KLF2 expression and clinicopathological parameters. Pearson or Spearman tests were used to assess the association between the two variables. All experiments were repeated three times. Experimental data were analyzed with GraphPad Prism 8.0 (GraphPad-La Jolla, CA, USA) software. Experimental results are presented as mean ± standard deviation (SD). Differences between the groups were analyzed using the paired Student's *t*-test or one-way analysis of variance (ANOVA) with the multiple group Tukey's multiple comparison post hoc test. *P* < 0.05 was considered statistically significant.

## 3. Results

### 3.1. KLF2 Is Lowly Expressed in GC

**Figure 1:**
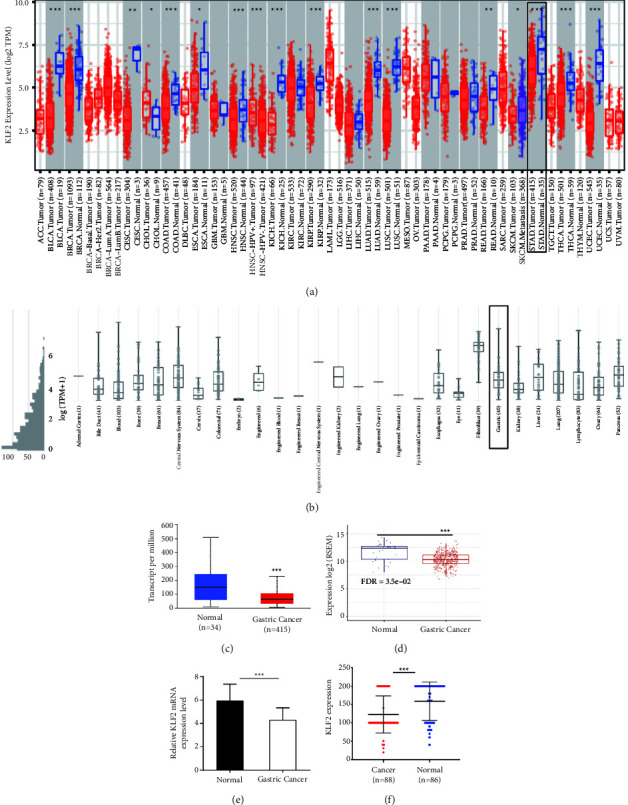
KLF2 expression is low in GC tissues. (a) TIMER2.0 database used to analyze mRNA levels of KLF2 in different tumor types. (b) KLF2 expression levels in different tumor cell lines detected by the CCLE database. (c) The TCGA database used to analyze KLF2 expression in GC tissues and paired adjacent noncancerous tissues. (d) The GEPIA database used to determine KLF2 expression in GC tissue and normal tissue data. (e) mRNA levels of KLF2 detected by RT-qPCR in GC tissues and paired adjacent noncancerous tissues (*n* = 30). (f) IHC used to analyze KLF2 protein levels in GC tissue microarrays. *P* < 0.05; *P* < 0.01;  ^*∗∗∗*^*P* < 0.001.

Aberrant expression of KLF2 has been reported in a variety of cancers [30, 31]; however, the association and the role of KLF2 and GC have not been elucidated. In this study, we found that KLF2 mRNA levels were KLF2 expression promotes the malignant biological behavior in GC cells and accelerates GC progression. lower in various tumor tissues than in normal tissues through the TIMER2.0 database, and KLF2 mRNA expression was significantly lower in STAD tissues than in normal tissues (Figure 1(a)). CCLE database analysis showed low KLF2 expression in GC cell lines (Figure 1(b)). Data from GC tissues downloaded from the TCGA database and GEPIA database for analysis were consistent with the above results (Figures 1(c) and 1(d)). Further validation by RT-qPCR analysis revealed a significant downregulation of KLF2 mRNA levels in GC patient tissues compared to adjacent normal gastric tissues (Figure 1(e)). In addition, immunohistochemical staining showed significantly higher KLF2 protein expression in adjacent normal gastric tissues than in GC tissues (Figure 1(f)). Together, these results suggest that KLF2 is aberrantly expressed in GC tissues and may be a potential target for GC and deserves in-depth investigation.

### 3.2. Association of KLF2 Genomic Alterations with Prognosis in GC

**Figure 2:**
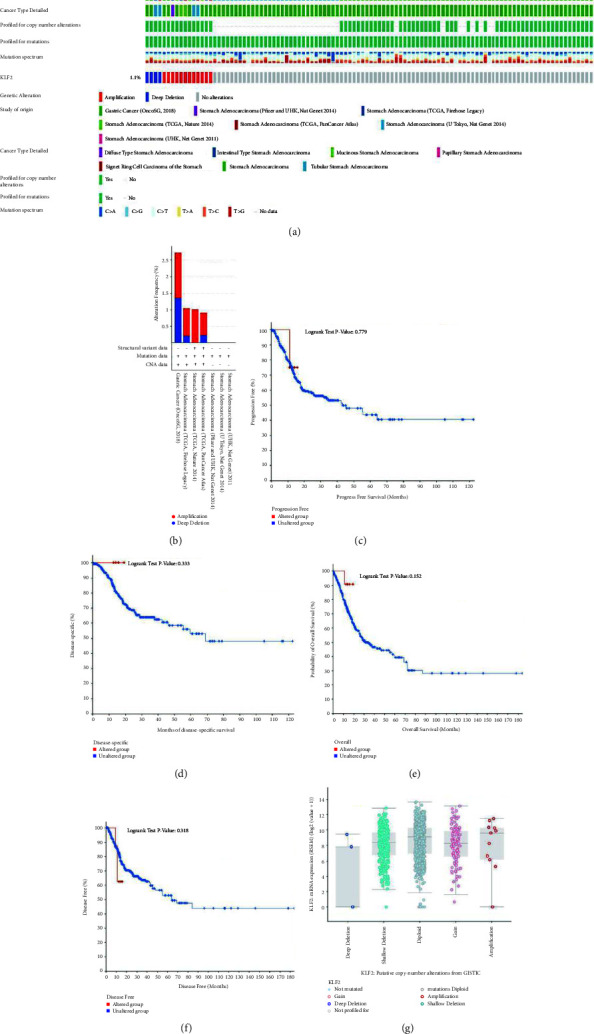
Association of KLF2 genomic alterations with prognosis in GC (cBioPortal). (a) OncoPrint genetically altered for KLF2 in GC cohort. (b) Cancer types summary of KLF2 genomic alterations in GC. Association of KLF2 mutations with disease-free survival (c), disease-specific survival (d), overall survival (e), and progression-free survival (f) in GC patients. (g) Box plots of KLF2 copy number changes (CNV) in GC for GISTIC. Hete., heterozygous; *Homo*., homozygous; Amp., amplification; Dele., deletion.

**Figure 3:**
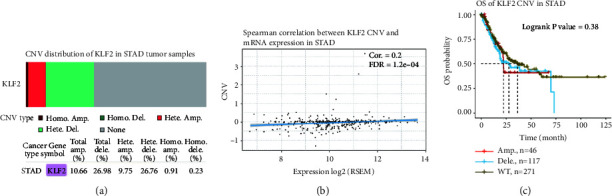
CNV analysis of the KLF2 gene in GC and its effect on overall survival (GSCA). (a) Proportion of KLF2 heterozygous/homozygous and amplified/deleted in STAD. (b) Spearman correlation assessment of CNV and KLF2 mRNA expression in STAD. (c) Survival difference between KLF2 CNV and wild type in STAD patients.

In order to elucidate the mechanisms underlying aberrant KLF2 expression, in this study, we used the cBioPortal database to analyze the amplification frequency and mutation types of KLF2 alterations in a cohort of GC patients. The results showed that KLF2 was mutated at a frequency of 1.1% in GC, and the mutation types included mutation and amplification (Figures 2(a) and 2(b)). Further analysis of KLF2 gene mutations on the prognosis of GC patients was performed. The results showed that GC patients in the KLF2 mutant group had poorer disease-free survival (Figure 2(c)), disease-specific survival (Figure 2(d)), overall survival (Figure 2(e)), and progression-free survival (Figure 2(f)) compared with the nonmutant group, but there was no significant difference (*P* > 0.05). In addition, we analyzed the CNV distribution (including amplification and deletion) of KLF2 in GC (Figure 2(g)). In addition, further analysis by the GSCA database showed 10.66% and 26.98% CNV for total amplification and total deletion of KLF2 in STAD, with 26.76% heterozygous deletions (Figure 3(a)). The Spearman correlation between KLF2 mRNA expression and CNV was 0.2 (Figure 3(b)). Overall survival (OS) was lower in KLF2 CNV (amplifications and deletions) compared to wild type (Log-rank *P* value = 0.38) (Figure 3(c)). These results suggest that low KLF2 expression in GC may be associated with genomic deletions.

### 3.3. Correlation between KLF2 Expression and Clinicopathologic Features of GC Patients

**Figure 4:**
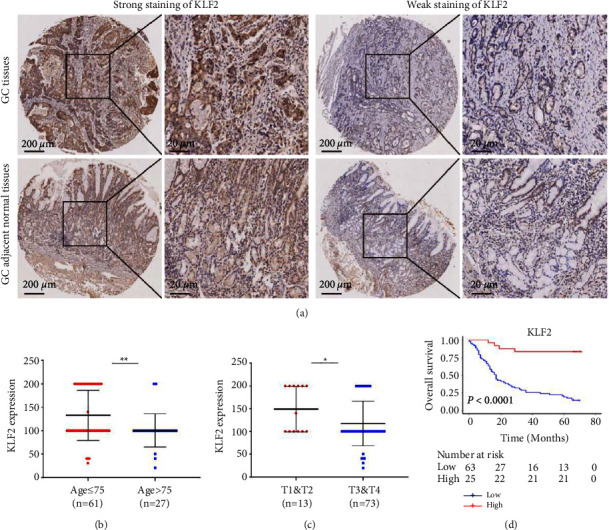
KLF2 protein expression in GC tissue microarrays (immunohistochemistry). (a) KLF2 protein expression in GC tissues (*n* = 88) and adjacent normal tissues (*n* = 86) (magnification: 200× and 400×). (b) KLF2 expression levels in GC patients of different age groups. (c) KLF2 protein expression in GC patients with different T stages. (d) Kaplan–Meier curves used to analyze KLF2 expression levels with overall survival in GC patients (log-rank test, *P* < 0.0001). ^*∗*^*P* < 0.05 ^*∗∗*^*P* < 0.01

Subsequently, KLF2 protein expression in GC tissue microarrays was detected by IHC in this study (Figure 4(a)), and the results showed that the higher the age of patients (>75 years) and the greater the T stage (T3 and T4), the lower the KLF2 expression level (Figures 4(b) and 4(c)). In addition, GC patients with low KLF2 expression had shorter survival times and lower overall survival (Figure 4(d)). Therefore, these results suggest that low KLF2 expression promotes tumor invasion/metastasis and can be used as a diagnostic biomarker for GC with an important clinical value.

### 3.4. Knockdown of KLF2 Promotes Malignant Biological Behavior of GC Cells

**Figure 5:**
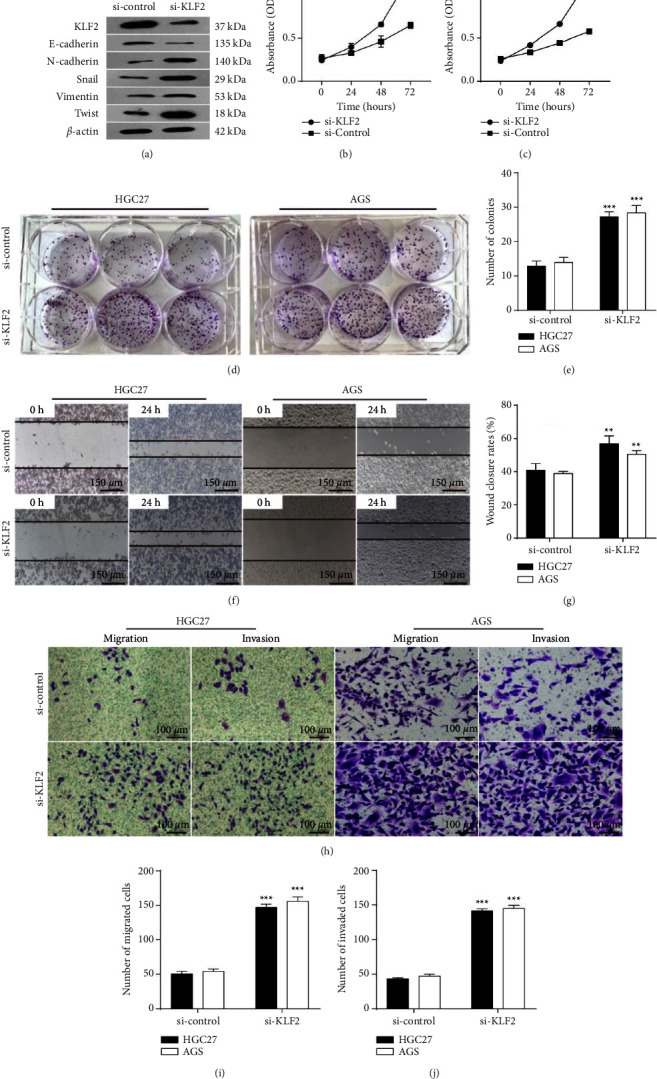
Knockdown of KLF2 promotes the growth, proliferation, migration, and invasion of GC cells. (a) Western blot used to analyze E-cadherin, N-cadherin, Snail, vimentin, and Twist protein expression levels in HGC-27 cells knocked down for KLF2, after KLF2 knockdown in HGC-27 and AGS GC cell lines. (b-c) Cell viability measured by CCK-8 assay. (d-e) A colony formation assay used to analyze GC cell proliferation ability. (f-g) Wound healing assay used to assess the migration ability of GC cells. (h–j) Transwell assay used to analyze migration and invasion of GC  ^*∗*^*P* < 0.05 ^*∗∗*^*P* < 0.01 ^*∗∗∗*^*P* < 0.001.

To further elucidate the mechanism of KLF2's action on GC, in this study, we investigated the effect of KLF2 on GC cell growth and proliferation, migration, and invasion by knocking down KLF2 in HGC-27 and AGS cells. Western blot analysis showed that knockdown of KLF2 in GC cells resulted in higher levels of N-cadherin, Snail, vimentin, and Twist protein expression than in the si-control group, but reduced E-cadherin protein expression (Figure 5(a)). CCK-8 and colony formation assays showed that knockdown of KLF2 significantly promoted HGC-27 and AGS cell growth and proliferation compared to the si-control group (Figures 5(b)–5(e)). In addition, wound healing assay and transwell analysis showed that the wound healing rate and number of migrated and invaded cells in the si-KLF2 group were significantly higher than those in the si-control group (Figures 5(f)–5(j)). These results indicate that low KLF2 expression promotes the malignant biological behavior in GC cells and accelerates GC progression.

## 4. Discussion

GC is one of the most common gastrointestinal tumors in mammals. Due to western diets, bad living habits, and increased pressure to study and work, the incidence of GC is rising,, which will cause a huge medical burden on society. It is reported that the occurrence of GC is closely related to many factors such as *Helicobacter pylori* infection, eating habits, environment, genetics, and immunity [32–34], among which genetic factors play a crucial role [35]. At present, the treatment of GC mainly includes drug and surgical treatment, but most patients still have metastasis and recurrence after conventional radiotherapy and chemotherapy [36]. In addition, many studies have focused on the important impact of host genetic susceptibility on the development of GC. Based on this, some abnormally expressed genes have been identified as biomarkers and therapeutic targets. However, the diagnosis and prognosis of GC are still not accurate. In this study, the expression mechanism, prognostic value, and potential role of KLF2 in GC development were investigated in depth by using bioinformatics techniques combined with clinical tissue samples and cellular experiments.

Cancer-associated inflammation emerging at different stages at the time of tumorigenesis has been reported to lead to genomic instability and epigenetic modifications [37]. Previous studies have shown that KLF2 is downregulated in GC and prone to genomic dysregulation [15]. In this study, KLF2 was significantly lower expressed in GC tissues and cells and may be associated with mutations in the KLF2 gene. Correlation analysis of clinical characteristics showed a negative correlation between KLF2 and age, T stage, and survival in GC patients. The knockdown of KLF2 in GC cells was further analyzed to investigate the effect of low KLF2 expression on GC function. The results showed that the knockdown of KLF2 could significantly promote the growth, proliferation, migration, and invasion of GC cells, as well as tumor development. This is in agreement with Wang et al. [38]. In addition, Li et al. showed that the long noncoding RNA DLEU1 promotes GC progression by epigenetically inhibiting KLF2 [39]. Another study showed that microRNAs promote GC cell proliferation and invasion by targeting KLF2 [16]. MicroRNA-32-5p promotes GC development by activating the PI3K/AKT signaling pathway and targeting KLF2 expression [40]. Furthermore, a study has shown that SUZ12 may regulate proliferation and metastasis of GC, through modulating KLF2 and E-cadherin [22]. We also observed a regulatory relationship between KLF2 and E-cadherin. The knockdown of KLF2 resulted in a reduction in E-cadherin. Furthermore, the expression of epithelial-mesenchymal transition (EMT)-related proteins (N-cadherin, Snail, Vimentin, and Twist) was increased after KLF2 knockdown. These studies suggest that KLF2 may be a novel therapeutic target for GC.

In conclusion, low KLF2 expression promotes the malignant biological behavior of GC, and KLF2 acts as a tumor suppressor and may be a potential therapeutic target and prognostic biomarker for GC. Although this study provides a rationale for the role of KLF2 in GC pathogenesis, we did not further investigate the effect of epigenetic alterations on KLF2 underexpression in GC nor did we assess the number of patients who developed KLF2 underexpression in GC, and therefore, we will continue in-depth studies through databases and clinical trials in the future.

## Data Availability

The data used to support the findings of this study are available from the corresponding author upon request.
